# Supplementary material for: OGR1 (GPR68) and TDAG8 (GPR65) Have Antagonistic Effects in Models of Colonic Inflammation
Source: Int J Mol Sci. 2023 Oct 3;24(19):14855. doi: 10.3390/ijms241914855 (PMC10573511; doi:10.3390/ijms241914855)

Supplementary Figure S1: The absence of OGR1 reduces inflammation upon DSS-induced acute colitis. A

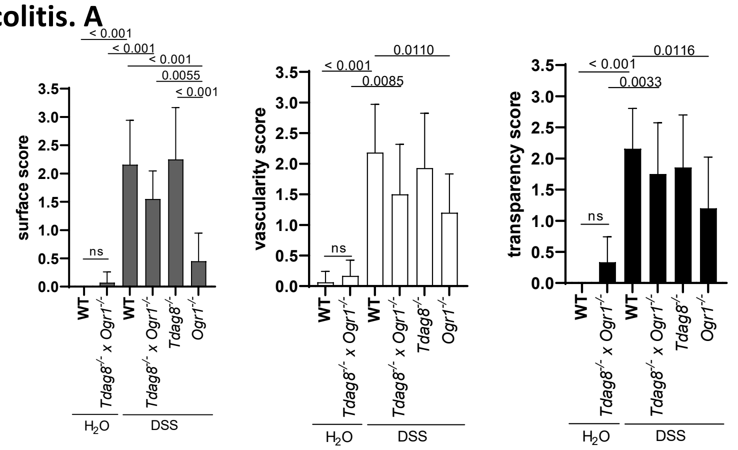

B

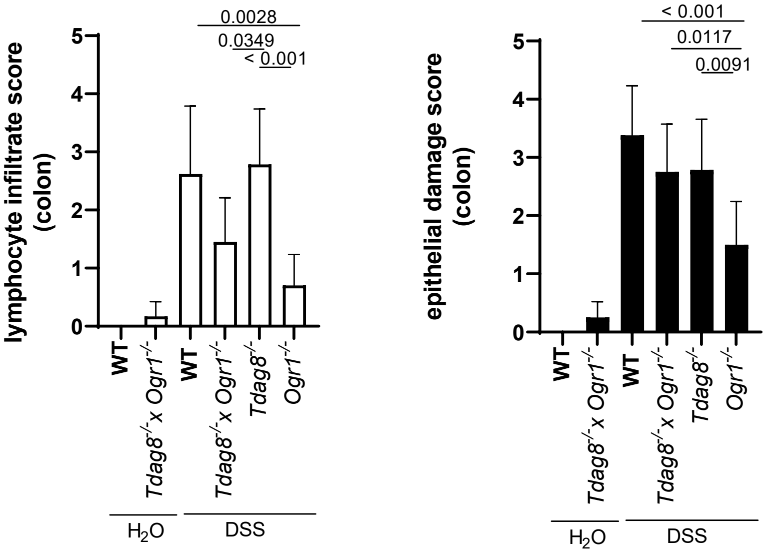

Supplementary Figure S2: The absence of TDAG8 aggravates inflammation in the chronic colitis model. A

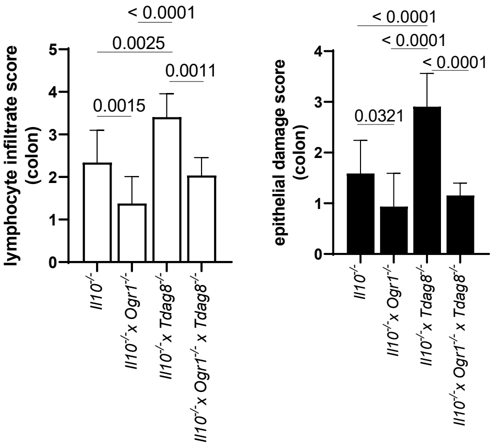

B

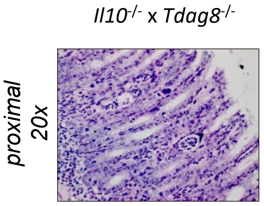

Supplementary Figure S3: Absence of TDAG8 aggravates inflammation in small bowel in the spontaneous colitis model.

A

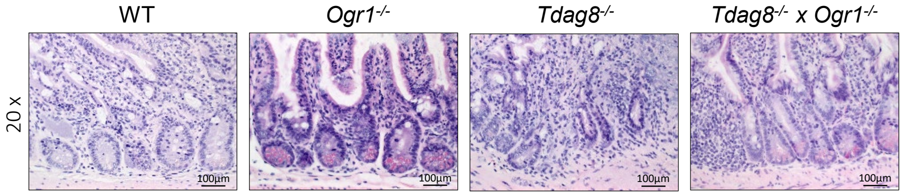

B

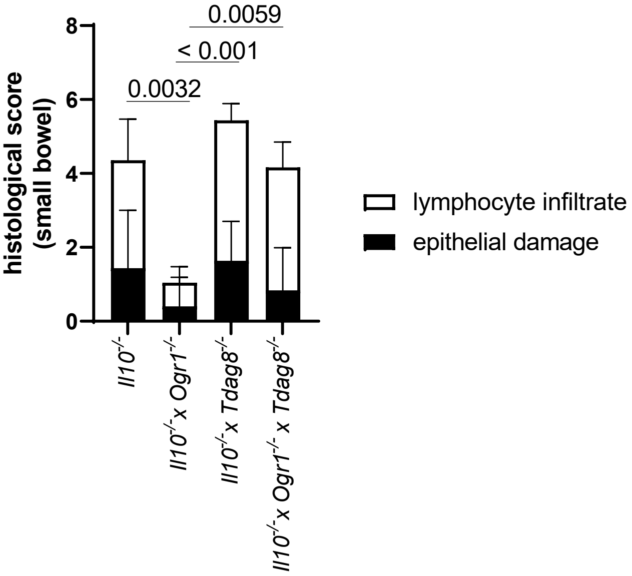

Supplementary Figure S4: FACS, manual gating from cells isolated from colon. A

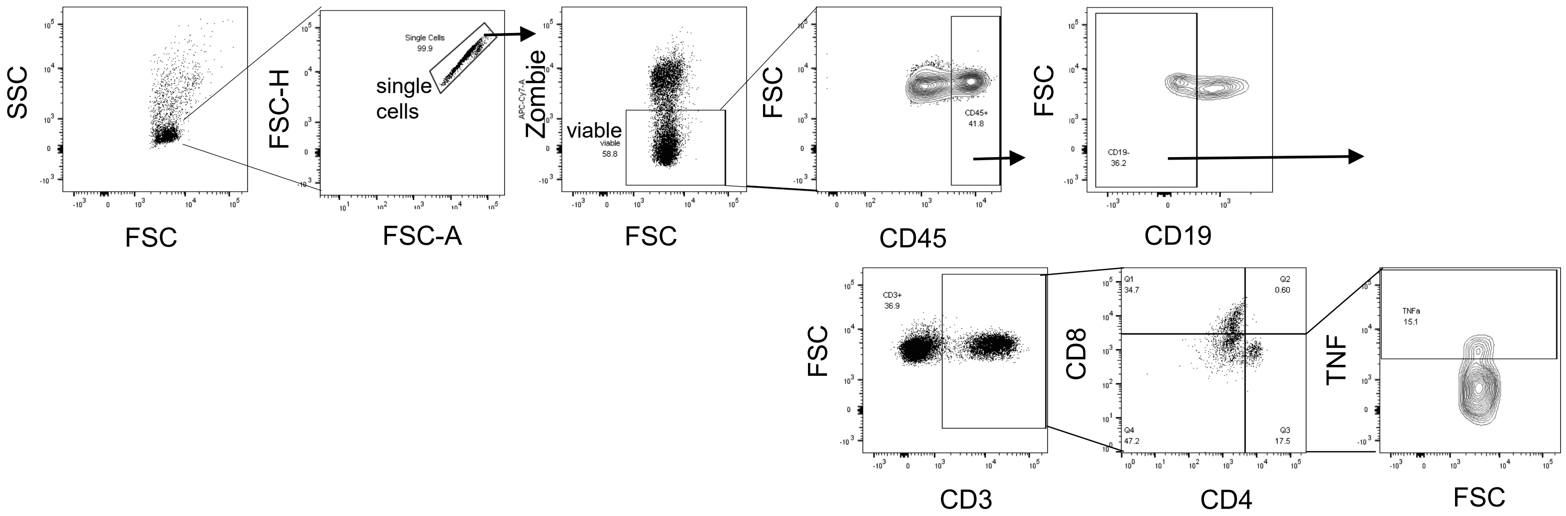

B

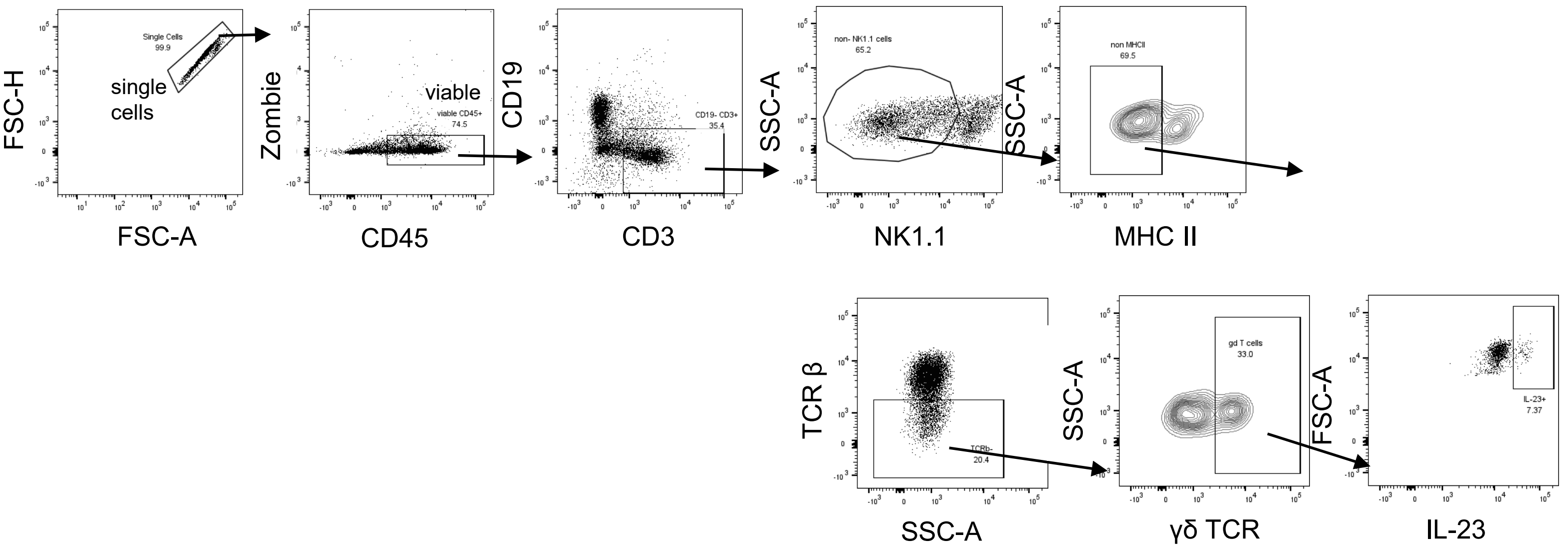

C

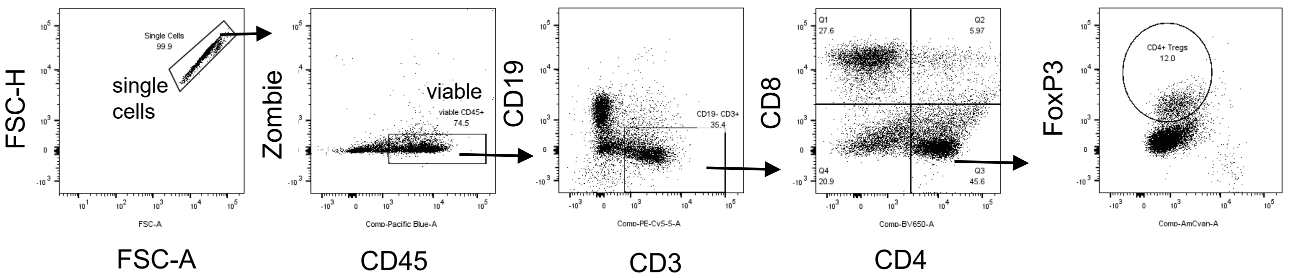

D

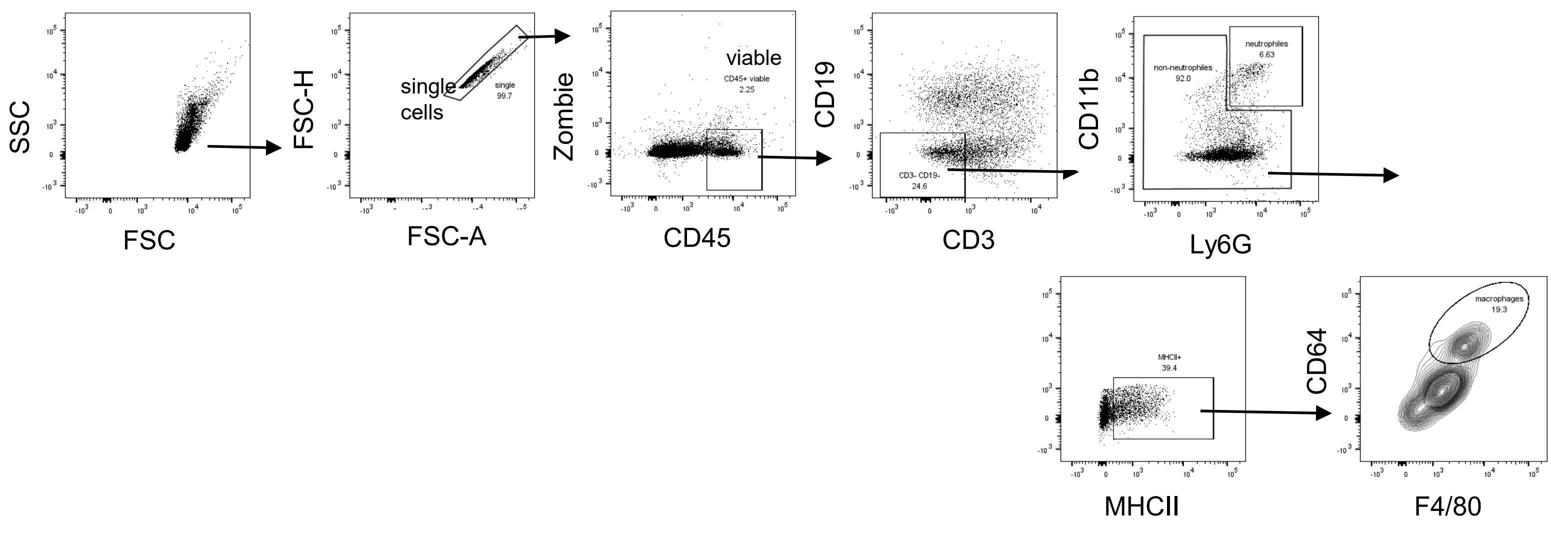

Supplementary Figure S5: FACS.

A

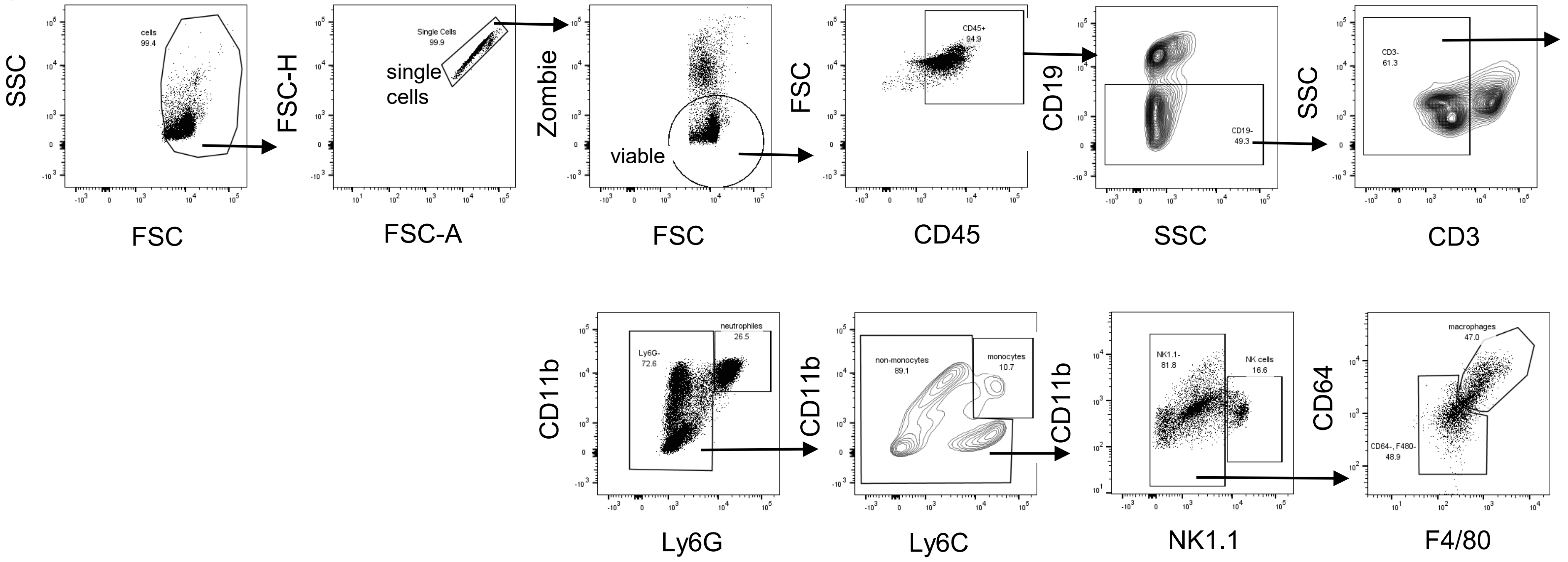

B

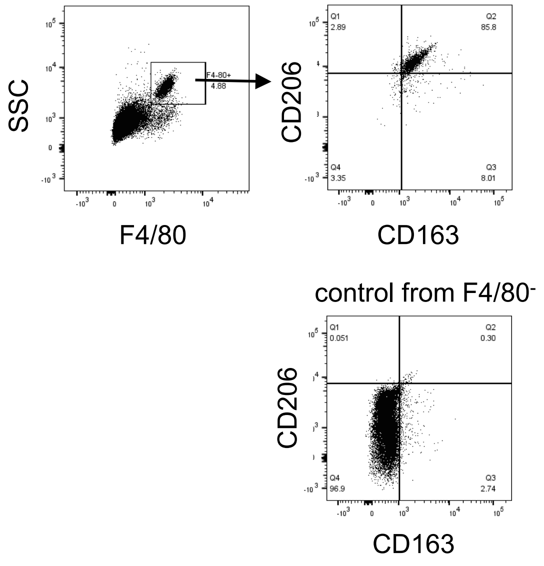

C

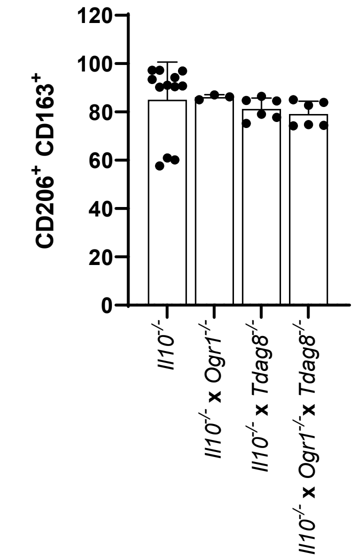

D

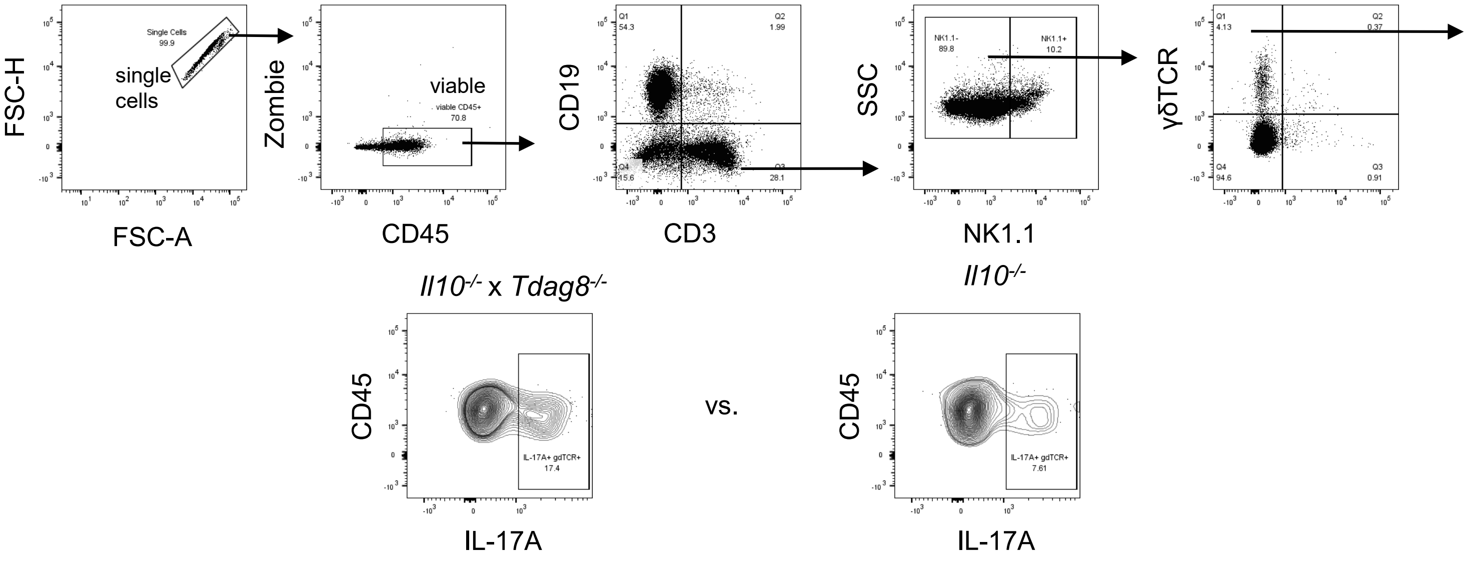

Supplement: Supplementary file 1 [file ijms-24-14855-s001.zip › ijms-2616429-supplementary Figures.pdf]
