# Supplementary material for: OGR1 (GPR68) and TDAG8 (GPR65) Have Antagonistic Effects in Models of Colonic Inflammation
Source: Int J Mol Sci. 2023 Oct 3;24(19):14855. doi: 10.3390/ijms241914855 (PMC10573511; doi:10.3390/ijms241914855)
Supplement: Supplementary file 1 [file ijms-24-14855-s001.zip › ijms-2616429-supplementary Table.pdf]

Supplementary Table

Table S1. Antibodies used for FACS (M = mouse; R = rat; H = hamster; NA = not applicable).

Fluorochrome channel	Target	Manufacturer	Reference number	Dilution	Host
**BV785**	TNFα	BioLegend	506341	1:200	R
**BV785**	NK1.1	BioLegend	108749	1:200	M
**BV711**	Ly6C	BioLegend	128037	1:200	R
**BV650**	CD4	BioLegend	100546	1:200	R
**BV650**	CD206	BioLegend	141723	1:200	R
**BV605**	CD11b	BioLegend	101257	1:200	R
**BV510, AmCyan**	Ly6G	BioLegend	127633	1:200	R
**BV510, AmCyan**	IL-17A	BioLegend	506933	1:200	R
**PacificBlue**	CD45	BioLegend	103126	1:400	R
**PE-Cy7**	$\gamma\delta$TCR	BioLegend	118124	1:200	H
**PE-Cy5.5**	CD3	Invitrogen	35-0031-82	1:200	H
**PE-Cy5**	B220	Invitrogen	15-0452-83	1:200	R
**FITC**	F4/80	eBioscience	11-4801-85	1:200	R
**PE-Texas Red**	CD8a	BD Biosciences	562283	1:400	R
**PE, DsRed**	FOXP3	BioLegend	320007	1:200	M
**PE, DsRed**	CD163	ThermoFisher	12-1631-82	1:100	R
**PE, DsRed**	CD64	BioLegend	139304	1:100	M
**APC-Cy7**	viability marker	BioLegend	423106	1:200	NA
**AF700**	MHC II	BioLegend	107622	1:200	R
**APC, AF647**	CD19	BioLegend	152410	1:200	R
